# Supplementary material for: Drug repurposing for aging research using model organisms
Source: Aging Cell. 2017 Jun 16;16(5):1006–15. doi: 10.1111/acel.12626 (PMC5595691; doi:10.1111/acel.12626)
Supplement: Supplementary file 7 — Data S1 Zip‐Archive of all report cards. [file ACEL-16-1006-s007.zip › RC_FRQ.pdf]

FRQ

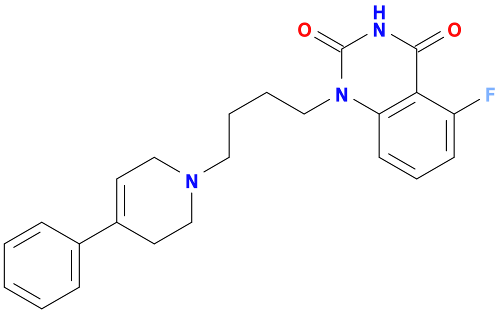

Database identifiers

ChEMBLCompound	CHEMBL361054
DrugBank	DB07787

Ranking

	Rank	Score
Drosophila	431/697	0.334
C. elegans	265/591	0.21

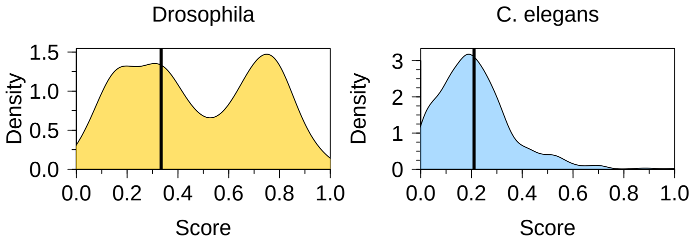

	Ageing implication	Domain conservation	Binding site conservation	Binding affinity	Bioavailability	Lipinski	Promiscuity	Purchasability	Drug approval	**Total**
Drosophila	0.36	0.927	0.918	0.941	(0.9)	0.0	-0.0	0.0	0.075	0.334
C. elegans	0.36	0.875	0.987	0.941	0.464	0.0	-0.0	0.0	0.075	0.21

Names

- 5-fluoro-1-4-(4-phenyl-3,6-dihydro-2H-pyridin-1-yl)butylquinazoline-2,4-dione
- 5-fluoro-1-4-(4-phenyl-3,6-dihydro-2H-pyridin-1-yl)butylquinazoline-2,4-quinone
- 5-fluoro-1-4-(4-phenyl-3,6-dihydropyridin-1(2h)-yl)butylquinazoline-2,4(1h,3h)-dione

Roles

ChEBI entry None has no roles

Status

Approved drug (according to ChEMBL)	No
Classification (according to DrugBank)	experimental
Number of Rule of 5 violations	0
Binding affinity to original target in log units (RF-Score prediction)	7.78
Burns *C. elegans* bioavailability prediction	2.4

Compound Target Characteristics

Poly [ADP-ribose] polymerase 1

Best gene implication in ageing for this target family came from gene Q921K2 via mapping the annotation from Ensembl ENSMUSG00000026496 via mapping the annotation from EntrezGene 11545 via mapping the annotation from GenAgeModels 1010 annotated in GenAge release 17.

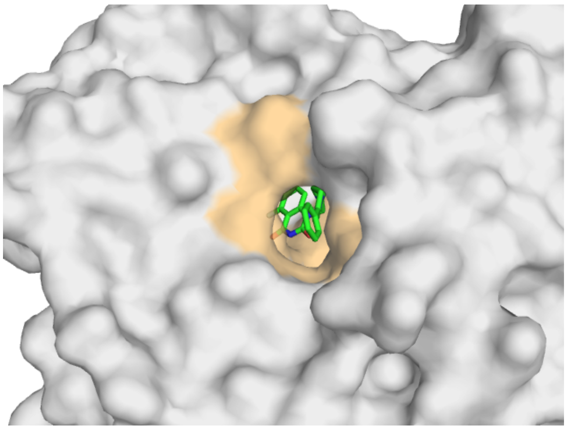

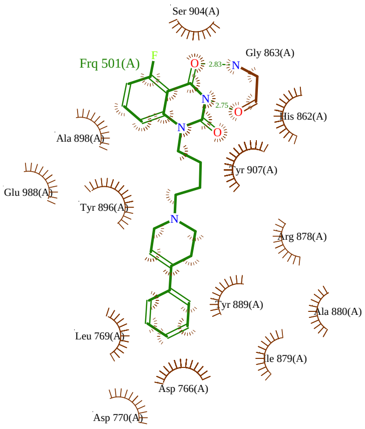

protein	amino acids contacts (binding site)
PDB:1uk1:chainA:P09874	D L D W H G R I A Y Y F A S Y E
sp:P09874:PARP1_HUMAN	D L D W H G R I A Y Y F A S Y E
sp:P27008:PARP1_RAT	D L D W H G R I A Y Y F A S Y E
tr:Q921K2:Q921K2_MOUSE	D L D W H G R I A Y Y F A S Y E
sp:P35875:PARP_DROME	D A E W H G R I A Y Y F A S Y E
sp:Q9N4H4:PME1_CAEEL	N L D W H G R I A Y Y F A S Y E

	whole protein		domain-based		contact-based	
protein	ident	simil	ident	simil	ident	simil
PDB:1uk1:chainA:P09874	1.0	1.0	1.0	1.0	1.0	1.0
sp:P09874:PARP1_HUMAN	1.0	1.0	1.0	1.0	1.0	1.0
sp:P27008:PARP1_RAT	0.92	0.97	0.95	0.99	1.0	1.0
tr:Q921K2:Q921K2_MOUSE	0.92	0.98	0.96	0.99	1.0	1.0
sp:P35875:PARP_DROME	0.41	0.77	0.54	0.85	0.88	0.92
sp:Q9N4H4:PME1_CAEEL	0.29	0.63	0.44	0.8	0.94	0.99

Parp (FBgn0010247) associated phenotypes

aging defective, heat sensitive, heat stress response defective, immune response defective, increased cell death, lethal - all die before end of pupal stage, non-enhancer of variegation, non-suppressor of variegation, planar polarity defective, some die during pupal stage

(Information from FlyBase)

Parp (UniProt:P35875) annotation

Function: Poly[ADP-ribose] polymerase modifies various nuclear proteins by poly(ADP-ribosyl)ation. The modification is dependent on DNA and is involved in the regulation of various important cellular processes such as differentiation, proliferation, and tumor transformation and also in the regulation of the molecular events involved in the recovery of cell from DNA damage. Plays a fundamental role in organizing chromatin on a global scale; isoform e autoregulates Parp transcription by influencing the chromatin structure of its heterochromatic environment. (PubMed:12183365).
Subcellular location: Nucleus ECO:0000255—PROSITE- ProRule:PRU00264, PubMed:12183365). Nucleus, nucleolus (PubMed:12183365). Note=Highly enriched in nucleoli, heterochromatic chromosomal regions, and diverse euchromatic sites in the cells of most embryonic and adult tissues.
Tissue specificity: Expressed in adult female oocytes, anal plates of stage 12 embryos and in cells around the central nervous system in later embryos. (PubMed:9565614).
Developmental stage: Expressed both maternally and zygotically in embryos, pupae and adults.

Expression is highest in embryos. (PubMed:12183365, PubMed:9565614).
Miscellaneous: The ADP-D-ribosyl group of NAD(+) is transferred to an acceptor carboxyl group on a histone or the enzyme itself, and further ADP-ribosyl groups are transferred to the 2'-position of the terminal adenosine moiety, building up a polymer with an average chain length of 20-30 units.

(Information from UniProt)

pme-1 (UniProt:Q9N4H4) annotation
Function: Poly[ADP-ribose] polymerase modifies various nuclear proteins by poly(ADP-ribosyl)ation, a post-translational modification synthesized after DNA damage that appears as an obligatory step in a detection/signaling pathway leading to the reparation of DNA strand breaks and programmed cell death. Involved in protection of the genome against mutations, probably via some participation in DNA repair. (PubMed:12600937).
Subcellular location: Nucleus ECO:0000305.
Developmental stage: Predominantly expressed at early embryonic stages and later in L4 and adult stages. (PubMed:12145714).

(Information from UniProt)
